# Lawrence Kohlberg’s Suicide: An Analysis of His Final Life Stage

**DOI:** 10.5964/ejop.17971

**Published:** 2025-11-28

**Authors:** Vikki Botes, Roelf van Niekerk

**Affiliations:** 1 Industrial and Organisational Psychology, Nelson Mandela University, Gqeberha, South Africa; University of Johannesburg, Johannesburg, South Africa

**Keywords:** psychobiography, Lawrence Kohlberg, Bronfenbrenner’s Bioecological Theory of Human Development, Bronfenbrenner’s Proximal-Person-Context-Time Model, career success stage

## Abstract

**Objective:**

This article focuses on the last stage of Kohlberg’s life, his career success years, between ages of 41 to 59 years. The aim of the study was to explore and describe how an accomplished moral development expert reached the point of taking his own life. The objectives of this article were to illuminate Kohlberg’s life story through (a) formulating a comprehensive and contextualised description of Kohlberg’s last stage of his life and (b) interpreting part of Kohlberg’s life story according to Bronfenbrenner’s Bioecological Theory of Human Development Framework.

**Method:**

The psychobiographical analysis used a qualitative paradigm, longitudinal single-case research design from a descriptive-interpretive approach. Bronfenbrenner’s Bioecological Theory of Human Development, incorporating its enmeshed, most recent scientific research design, the Proximal-Person-Context-Time Model was used as the theoretical framework.

**Results/Findings:**

The findings illustrate the direct influence of the Context, Person and Time components on Kohlberg’s Proximal processes’ functioning levels and their outcomes. Kohlberg’s Proximal processes were impacted by several areas. These were his interpersonal relationships, career, challenges versus protective factors and visionary qualities.

**Conclusion:**

This article underscores the critical importance of suicide awareness and advocates for a comprehensive, holistic approach to addressing mental health challenges. It also stresses the need to investigate intricate and multifaceted factors that can influence suicidal behaviour, along with identifying protective factors that may help prevent suicidal ideation and intent. Understanding the complex factors that contribute to the risk of suicide allows for the effective identification and management of challenges, ultimately supporting greater mental health and well-being.

According to the World Health Organization (2024), more than 720,000 deaths occur on an annual basis due to suicide. Suicide can occur at any stage of life, and it usually has a lasting impact on significant others in the individual’s life. Suicide is not driven by an isolated event. It is often driven by multiple factors, such as adverse life events, mental health, cultural and social factors. Some suicides are impulsive acts, taking place during a crisis such as relationship difficulties, financial challenges, illness and chronic pain. In 2022, 49,449 individuals committed suicide in the United States of America (USA). This number increased by 2.6% since 2021, when 48183 individuals committed suicide (Substance Abuse and Mental Health Services Administration, 2023).

With the high prevalence of suicide rates, this article seeks to destigmatize suicide and highlight the importance of incorporating a more holistic approach when assisting clients in overcoming crises. Kohlberg’s psychobiography (Botes, 2022) illuminates the importance of understanding the complex factors associated with the increased risk of suicide, thereby enabling more effective identification and management of challenges which can assist in the prevention of suicide. This article, based on a doctoral study, focuses on Kohlberg’s last stage of his life, his career success years (1968–1987), and emphasises the importance of addressing and working through challenges in various areas of life to create more balanced functioning (Botes, 2022).

The application of Bronfenbrenner’s Bioecological Theory of Human Development (BTHD) and the Proximal-Person-Context-Time model (PPCT) to Kohlberg’s career success years allows for a multilayered and multidimensional exploration and description of those years. The PPCT model was developed by Bronfenbrenner to operationalise the Bioecological Theory of Human Development, thereby not only increasing the reliability and validity of the Bioecological Theory of Human Development but also increasing the multifaceted structure and complexity of this framework (Botes, 2022; Bronfenbrenner & Morris, 1998, 2006; Rosa & Tudge, 2013).

Due to the breadth of this theoretical framework, the depth of each facet is limited within the parameters of a single article. The focus in this article is on forming an understanding of how various facets interacted and preceded Kohlberg’s eventual suicide. Thus, this study is not only an in-depth application of the BTHD to Kohlberg’s career success years but also provides grounding for future research into how intricate systems interact in cases of suicidality, especially with high-functioning individuals.

## Overview of Kohlberg’s Life Story

Kohlberg was known for his forward-thinking research on moral development (Kohlberg, 1958, 1971, 1981, 1984; Snarey, 2012; Snarey & Samuelson, 2014). He achieved success in his career as a moral development specialist and teacher, yet he committed suicide in 1987 (Botes, 2022). Although this psychobiographical analysis focuses on Kohlberg’s last stage of his life namely, his career success years, a brief overview of Kohlberg’s life follows.

Kohlberg faced several challenges during his childhood and adolescent years. For example, he witnessed and experienced family turmoil and upheaval due to his parents’ divorce. Furthermore, Kohlberg and his siblings rotated between each parent every six months for five years (Fowler et al., 1988). Kohlberg rebelled against his own wealthy socio-economic status and his challenging childhood experiences by misbehaving at the private boarding school he attended, as well as how he chose to travel, who he interacted with during his travels and his physical appearance (Fowler et al., 1988; Garz, 2009).

After high school, Kohlberg wanted to study law or psychology. However, he became increasingly concerned with the horrors committed by the Nazis in World War II (WWII). Thus, he joined the United States Marines in 1945 instead (Snarey, 2012). After serving in the Marines, Kohlberg became a member of the Jewish Defence Force Haganah and worked aboard one of their ships. During Kohlberg’s time with the Haganah, he smuggled Jewish refugees from Europe into Palestine. He was eventually captured by the British forces and sent to a detention camp in Cyprus. After seven weeks in the detention camp, Kohlberg escaped to Palestine (Snarey, 2012). Kohlberg returned to the USA in 1948.

Due to Kohlberg’s experiences during WWII, he questioned how a cultured and educated nation such as Germany could approve of the Nazi reign. Therefore, after studying clinical psychology, Kohlberg focused on academic psychology, where he could explore morality. Kohlberg’s doctoral dissertation focused on moral development in children. This research brought Kohlberg to the attention of psychologists, and he gradually became known as one of the distinguished psychologists at the time (Snarey, 2012; Walsh, 2000).

This article focuses only on the last stage of Kohlberg’s life, namely his *career success* years between 1968 to 1987. The aim is to explore and describe the behaviours and events that preceded his suicide (Botes, 2022). The following section provides an overview on the approach and methods used in the study.

## Method

The aims were to illuminate Kohlberg’s life story through, (a) formulating a comprehensive and contextualised description of Kohlberg’s last stage of his life and, (b) interpreting part of Kohlberg’s life story according to Bronfenbrenner’s Bioecological Theory of Human Development (BTHD) Framework (Botes, 2022).

This psychobiographical analysis used a qualitative paradigm, longitudinal single-case research design from a descriptive-interpretive approach. The descriptive-interpretive paradigm approach allowed for the exploration of the informal applicability of Bronfenbrenner’s BTHD Framework to Kohlberg last life stage. The study used the theoretical framework of the BTHD, by including its most up-to-date scientific research design, the Proximal-Person-Context-Time Model (PPCT) (Botes, 2022; Bronfenbrenner & Morris, 2006).

The data collection process focused on publicly available sources. These included biographic and autobiographic material, books, newspapers and journal articles. The search for sources was conducted over an 18-month period using internet and academic database searches. These included databases such as the Directory of Open Access Journals (DOAJ), JSTOR, Google Scholar, Sabinet, and EBSCOhost. The assessment of the data trustworthiness was guided by Lincoln and Guba’s (1985) model of trustworthiness (Botes, 2022).

Lincoln and Guba’s (1985) model of trustworthiness criteria focus on four areas. Those are namely, credibility, transferability, dependability, and confirmability. Credibility was established through data triangulation to corroborate the information across sources. Data was considered to be accurate and trustworthy when the content in different sources of data corresponded during the data triangulation process, increasing the true value and trustworthiness of the data (Botes, 2022; Yin, 2018).

Transferability was achieved by providing rich descriptions to enable theoretical generalisation rather than statistical generalisation, consistent with psychobiographical methods (Botes, 2022; Mayer & Kőváry, 2019; van Niekerk, 2021). Dependability was reinforced through oversight by a supervisor who is an expert in psychobiographical research to ensure consistent interpretation of the data. Lastly, confirmability was ensured through two measures. These were prolonged engagement with data and using an independent reviewer to verify the accurate use of Alexander’s (1990) Two-Question Model, strengthening the study’s objectivity and internal validity (Botes, 2022; Yin, 2018).

Alexander’s (1990) Two-Question Model ensured relevancy of the data by providing criteria for data inclusion based on the aims of the study. The use of Alexander’s Two-Question Model allowed for the inclusion of suitable data, the discovery of new insights and exclusion of irrelevant data. Data collection was concluded once data saturation had been reached. To assess the study’s internal validity, an independent reviewer examined the sources to verify the accurate application of Alexander’s model (Du Plessis, 2017).

## Overview of Bronfenbrenner’s Bioecological Theory of Human Development

The BTHD Framework describes human development, action and behaviour from different facets. This framework, incorporating the PPCT Model, concentrates on important interactions between diverse systems in the development of individuals. These are individual characteristics, proximal processes (motivating drives for human development), context and time (Mc Guckin & Minton, 2014; Rosa & Tudge, 2013).

Bronfenbrenner’s theory was considered appropriate for this psychobiographical analysis as it is a *grand theory.* Therefore, it focuses on different aspects in individuals lives such as education, culture, intelligence, religion, personality and education instead of only focusing on one factor such as personality (Du Plessis, 2017). In Kohlberg’s psychobiography, the BTHD Framework allowed for the exploration and description of numerous factors present during his last stage. These included factors such as Kohlberg’s personality, family, cultural and historical background, health and vocation (Botes, 2022).

## Findings

Kohlberg’s development during the final stage of his life (1968–1987), his *career success* years, spanned from the ages of 41 to 59 years. Firstly, Kohlberg’s career success stage was explored within the *context* component, by describing the micro-, meso-, exo-, and macrosystems. This component explores how different environments interact with the individual in a reciprocal manner to shape the individual’s development (Bronfenbrenner, 1977). Secondly, the characteristics of force, resource characteristics and demand characteristics were presented within the *person* component. The person component focuses on individual characteristics at various levels, such as dispositions, abilities and competencies. Thirdly, the macrotime within the *time* component was explored. The macrotime explores historical and sociocultural influences on an individual. This was done by sub-dividing the macrotime into family origins and historical context (Botes, 2022; Bronfenbrenner & Morris, 2006). A discussion of each of these in Kohlberg’s last stage of life follows.

### The Context Component

**Figure 1:**
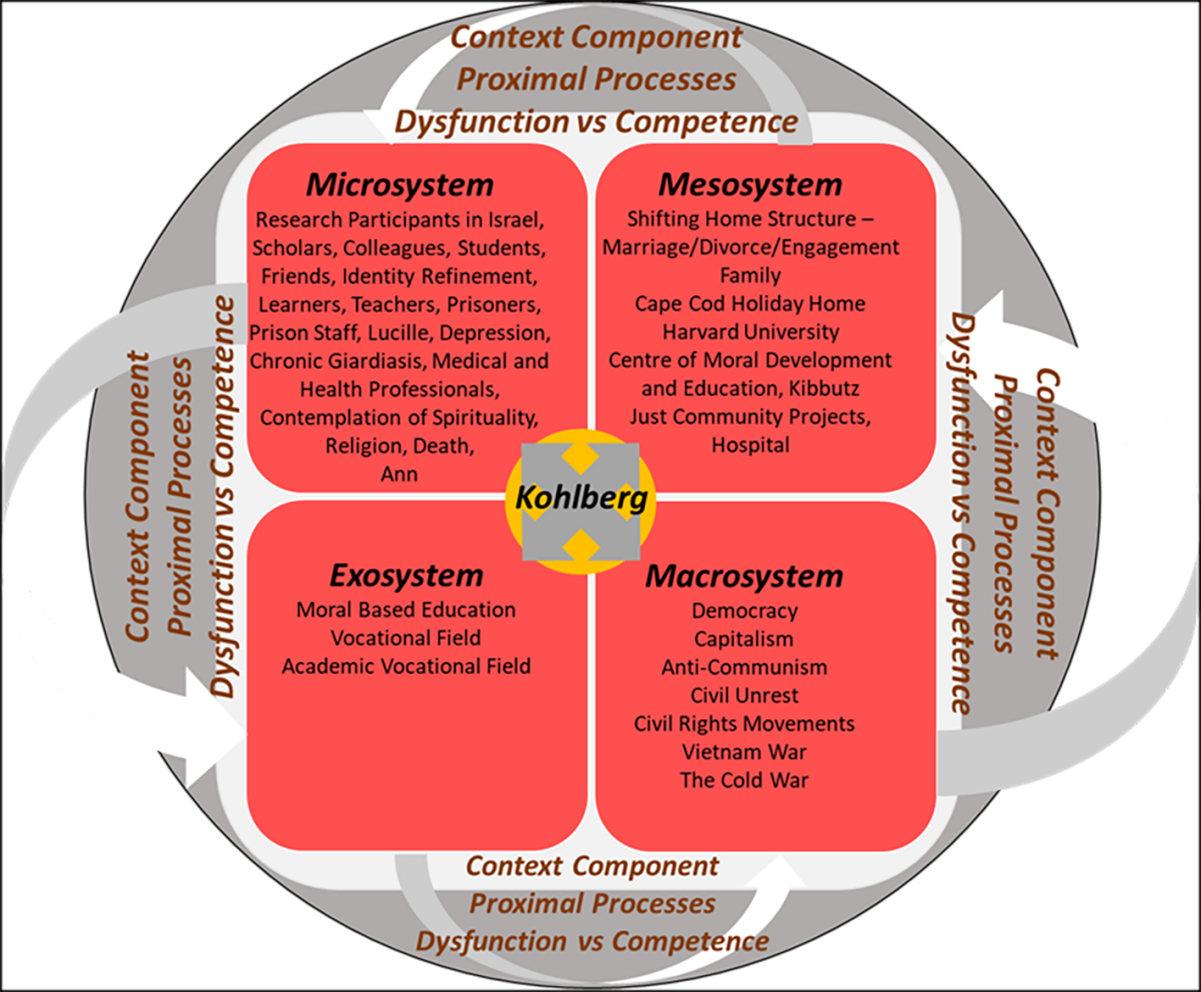
The BTHD — Career Success — The Context Component *Note*. Created from Bronfenbrenner and Morris (2006).

Visual depictions were created to illustrate the dynamic interplays of the elements in Kohlberg’s last stage of his life. This was achieved by using the last version of the BTHD Framework by combining the BTHD and the PPCT Model. Figure 1 shows the context components applicable to Kohlberg during his career success years stage (Botes, 2022; Bronfenbrenner & Morris, 2006).

The **microsystem** (Bronfenbrenner, 1977) in Kohlberg’s *career success* stage relates to the interactions he had with those within his academic sphere, his identity as an academic, researcher and moral educator, as well as his drive to assist humanity in a concrete and realistic approach (DeVries, 1991; Springer, 2005). Kohlberg’s behaviour and physical appearance were also noted to have developed into that of an academic (Walsh, 2000). Kohlberg interacted with students, colleagues, friends, scholars and critics (Bronfenbrenner, 1977; Snarey, 2012; Walsh, 2000).

Walsh (2000) explained that while Kohlberg became the authority on moral development, he did not only interact with people who promoted his research, but he also surrounded himself with his critics, becoming friends with many. Additional significant individuals within Kohlberg’s academic microsystem (Bronfenbrenner, 1977) were the participants in his research. This microsystem was deemed important to include in the findings, as the participants became an inspiration for Kohlberg to become a moral educator (Garz, 2009; Springer, 2005; Walsh, 2000).

Kohlberg’s personal life in his microsystem (Bronfenbrenner, 1977) included his mental and physical health due to contracting chronic *giardiasis*. It also included his interactions with his wife Lucille, his extended family, as well as his girlfriend and colleague Ann Higgins after his marriage to Lucille had ended (Snarey, 2012; Springer, 2005; Walsh, 2000). Snarey (2012), Springer (2005), and Walsh (2000) recalled that while Kohlberg experienced professional triumph, he suffered from depression due to his continuous gastric challenges and his deteriorating relationship with Lucille.

Although the literature explaining the reasons for Kohlberg and Lucille’s marriage disintegration is limited, their marriage was reported to be unhappy. They separated in 1974 and divorced 11 years later in 1985 (Snarey, 2012; Springer, 2005). During this time, the interactions of Kohlberg with his extended family were illuminated when Kohlberg’s sister Marjorie stated that despite his deteriorating health, Kohlberg remained a source of support for the family (Fowler et al., 1988). Due to Kohlberg’s chronic *giardiasis* and his depression, his microsystem (Bronfenbrenner, 1977) also evolved to include alternative health and medical professionals (Springer, 2005).

Although the literature does not discuss Kohlberg’s interactions with health and medical professionals, it is assumed that he would have interacted with them, when he suffered a nervous breakdown in 1979 and a suicide attempt in January 1987 (Garz, 2009; Higgins, 1991; Springer, 2005). Guided partially by his own suffering, towards the end of Kohlberg’s life, his microsystem (Bronfenbrenner, 1977) changed once more to self-exploration through religion and spirituality (Fowler et al., 1988; Snarey, 2012). Due to his imminent death, Kohlberg reflected on different questions such as: ‘How to face death?’ and ‘Why be just in an unjust world?’, Kohlberg found comfort in believing that we are a part of the cosmos which is continuously evolving and intrinsically lawful. Once this is understood, we acquire an awareness of moral relief (Higgins, 1991; Zizek et al., 2015).

The coinciding **mesosystem** elements from Kohlberg’s microsystem (Bronfenbrenner, 1977) were his changes in home structures. His home structures changed three times during this stage. It altered from a nuclear family during his marriage to Lucille, to a split family during his separation and divorce from Lucille in 1985. Then Kohlberg’s home structure changed once more when he dated and become engaged to a colleague, Ann (Springer, 2005). Other mesosystem elements were his extended family and the centre Kohlberg founded at Harvard. There was a negative interaction between Kohlberg’s physical health and his marriage to Lucille versus the Centre of Moral Development and Education at Harvard University (Bronfenbrenner, 1977; Snarey, 2012; Walsh, 2000).

The more successful Kohlberg’s career became, the more his marriage and health deteriorated (Bronfenbrenner, 1977; Snarey, 2012; Walsh, 2000). Other factors contributing to the decline of Kohlberg’s health and the deterioration of his marriage to Lucille was his demanding work schedule and his over-availability and sociability with people around him (Springer, 2005). Power (1991) explained that many people consistently queued to meet with Kohlberg. Despite Kohlberg’s declining health, he adhered to a demanding workload. His sister Marjorie begged him to take time off work, so that his health could improve (Fowler et al., 1988).

However, Kohlberg continued to sustain a demanding work schedule (Fowler at al., 1988; Higgins, 1991; Snarey, 2012). Other mesosystems were the hospital environment and the Kibbutz where he conducted his research, and the Just Community project. An additional environment noted within the mesosystem was Kohlberg’s holiday house at Cape Cod (Bronfenbrenner, 1977; Higgins, 1991; Kurtines & Gewirtz, 1991; Springer, 2005; Zizek et al., 2015).

Kohlberg’s **exosystem** (Bronfenbrenner, 1979) included the vocational fields of academia and moral-based education (DeVries, 1991; Kurtines & Gewirtz, 1991). Both of these had successes and setbacks. Due to Kohlberg’s success with his theory of moral development, he had international people come to meet him at the Centre for Moral Education and Development (Snarey, 2012; Walsh, 2000). An example of a setback Kohlberg encountered was to his professional reputation when it was discovered that moral development was bi-directional. This contradicted Kohlberg’s doctoral research findings claiming that it was unidirectional (Kohlberg, 1958; Springer, 2005; Zizek et al., 2015).

Kohlberg’s **macrosystem** (Bronfenbrenner & Morris, 2006) in the last stage of his life was made up of the economic, social and political systems of capitalism, democracy and anti-communism. Kuhmerker (1991) provided an illustration of how the macrosystem had a direct reciprocal influence on Kohlberg’s environment (Bronfenbrenner & Morris, 2006). This was shown though an account provided by Sprinthall in Kuhmerker (1991). Sprinthall explained that the faculty at Harvard University was angry over the Nixon-Kissinger invasion of Laos and Cambodia between 1969 to 1973. Despite this, people were reluctant to take the departments condemnation to the Nixon administration, however, Kohlberg volunteered for this (Kuhmerker, 1991).

In this section, the *context* components pertaining to Kohlberg’s last stage of life, his *career success* stage were presented (Bronfenbrenner, 1979). The next section explores the *person* components relevant to this stage of Kohlberg’s life (Bronfenbrenner & Morris, 2006).

### The Person Component

**Figure 2:**
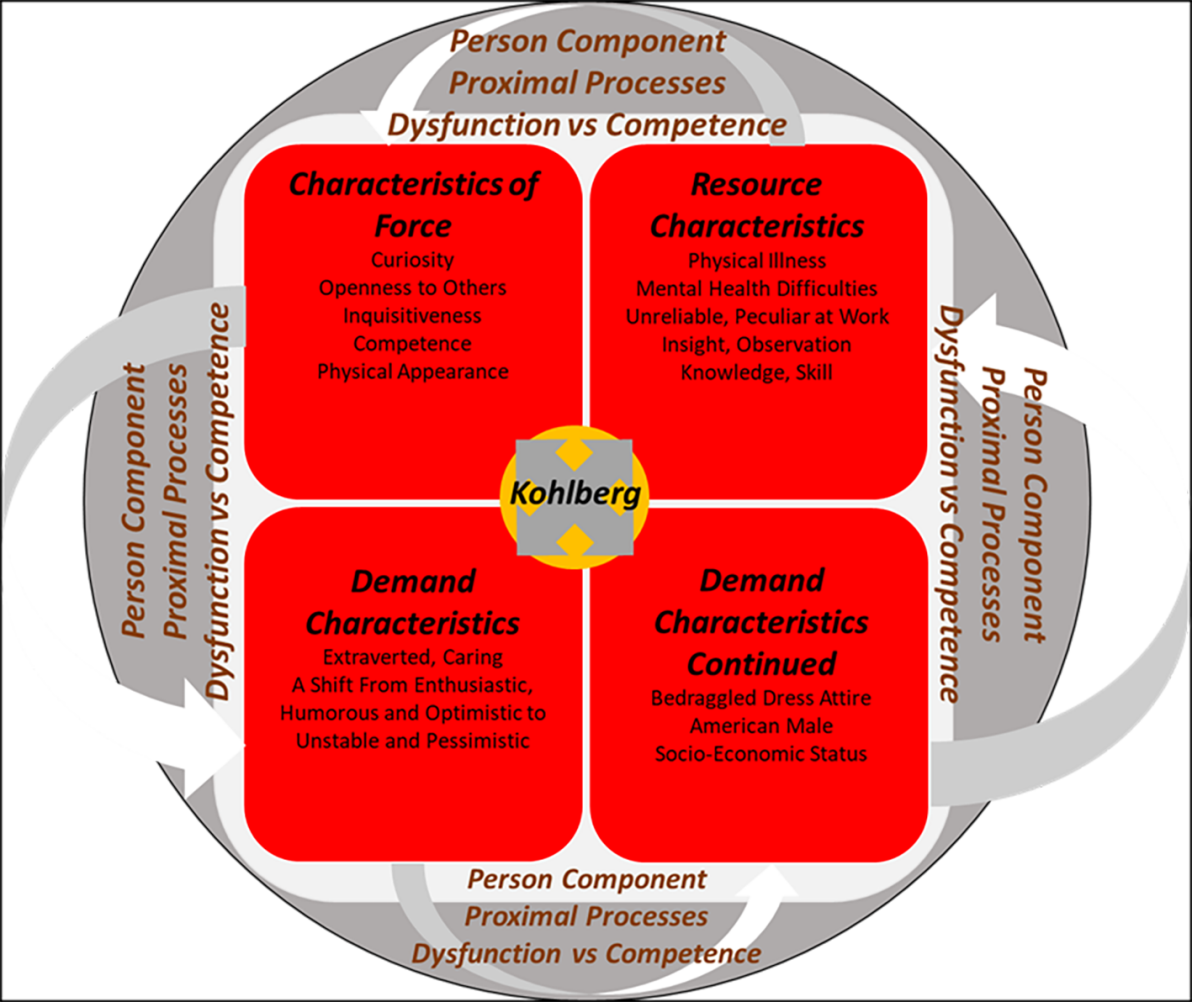
The BTHD — Career Success — The Person Component *Note*. Created from Bronfenbrenner and Morris (2006).

Figure 2 illustrates the person components relevant to Kohlberg during his career success years stage (Bronfenbrenner & Morris, 2006).

During this period, Kohlberg’s **characteristics of force** are identified as openness to others, curiosity and inquisitiveness. The negative characteristics of inattention and impulsiveness were also seen in some of Kohlberg’s behaviours such as when he continued to ram his car for a second time into the brick wall at the Larsen parking lot (Bronfenbrenner & Morris, 2006; Kuhmerker, 1991). Prior to Kohlberg contracting chronic *giardiasis*, he possessed the **resource characteristics** of observation, knowledge, insight and skills in his work environment (Bronfenbrenner & Morris, 2006).

However, after Kohlberg became ill in 1971/1972, he became known as being peculiar and unreliable at work. Kohlberg excused himself from meetings due to his painful physical symptoms (DeVries, 1991; Higgins, 1991; Snarey, 2012; Springer, 2005). A change in Kohlberg’s **demand characteristics** (Bronfenbrenner & Morris, 2006) was also evident when Marjorie confirmed the shift in Kohlberg’s demand characteristics from being an enthusiastic, amusing and optimistic individual to becoming an agitated, erratic and pessimistic individual (Fowler et al., 1988).

Kohlberg’s physical appearance was also highlighted through a story his friend Helkama shared. When Kohlberg arrived at his hotel in Finland, he saw a drunk man lying by the hotel entrance and tried to assist him. The doorman at the hotel saw this and assumed that Kohlberg was also homeless, due to the way he dressed (Zizek et al., 2015). This section within Kohlberg’s last stage of life, his *career success* stage, described the *context* and *person* components relevant to this stage. A discussion of the *time* components relevant to this stage follows (Bronfenbrenner & Morris, 2006).

### The Time Component

The time component within Kohlberg’s *career success* stage (from 1968 to 1987) is divided into two macrotime categories. Those are Kohlberg’s **family origins** and the **historical context**.

#### Macrotime — Family Origins

The first themes identified to have contributed to Kohlberg’s mental health during his career success years were the themes of **rebelliousness** and **fighting for a cause**. Kohlberg backed civil unrest, supporting the pursuit of independent thinking and justice. The cultural and political climate increased Kohlberg’s popularity within the USA (Carlisle, 2009; Rest et al., 1988). Another theme identified is the theme of **avoiding being a burden** on others. This was evident in this last stage of his life when Kohlberg attempted to commit suicide in early January 1987. During Kohlberg’s stay at Mount Auburn Hospital, Helkama (Zizek et al., 2015) recalled that Kohlberg continuously apologised to those around him for the trouble he was causing.

#### Macrotime — Historical Context

During Kohlberg’s career success stage beginning in 1968, the USA was still encountering substantial civil unrest, due to the country’s involvement in the Vietnam War. This caused a divide in public opinion, leading to violent protests in the runup race to the presidential election in 1968 as well as the assassination of prominent figures such as Robert Kennedy and Martin Luther King, Jr. However, when Nixon was re-elected in 1972, a truce was negotiated with North Vietnam (Carlisle, 2009; Gettleman et al., 1995).

Despite this, two decades of the Cold War, nuclear threats and fighting communism had a powerful effect on the daily lives of USA citizens. However, the USA economy continued to grow, leading to decreased unemployment rates and increased incomes. By the end of the 1970’s, the USA also advanced in its racial and gender equalities, as well as made gains in medicine, technology, science and further education (Carlisle, 2009; Fink, 2017; Gaddis, 2006; Gettleman et al., 1995).

## Discussion

Based on the findings of the context, person and time components during Kohlberg’s last stage, his career success stage, Kohlberg’s proximal processes and their outcomes are reviewed in relation to his mental health (Bronfenbrenner & Morris, 2006). These were his interpersonal relationships, career, challenges versus protective factors and visionary qualities. A discussion on each of these areas follows.

### The Proximal Processes Component

The first area explored during Kohlberg’s career success years was his **interpersonal relationships**. In Kohlberg’s microsystem he focused on establishing and maintaining friendships with students, colleagues, scholars and critics. Snarey (2012) explained that Kohlberg was eager to get involved in his students lives. This resulted in many of his students becoming his colleagues and friends. However, Kohlberg’s sociability and over accessibility to others also created an enmeshment between Kohlberg’s personal life and his vocation/academic life. After Kohlberg became sick with chronic *giardiasis*, his interpersonal relationships changed towards a dysfunctional outcome (Bronfenbrenner & Morris, 2006).

Kohlberg was perceived to have changed from possessing the demand characteristics of optimism, humour and enthusiasm to possessing the characteristics of pessimism, instability and irritability (Fowler et al., 1988). Kohlberg’s physical appearance in his characteristics of force, demonstrated how it formed his environment’s response to him within his microsystem (Bronfenbrenner & Morris, 2006). This was seen through Helkama’s story of Kohlberg in Finland (Zizek et al., 2015).

Despite Kohlberg’s appearance, his demand characteristic of caring stayed strong. According to Marjorie, Kohlberg was the person the extended family would turn to for support (Fowler et al., 1988). However, the demand characteristics of being unstable, pessimistic and frantic also impacted on the quality of the interactions between Kohlberg and his environment, shaping it to become more negative (Bronfenbrenner & Morris, 2006). Thus, it is assumed that Kohlberg’s professional triumphs and the demands it placed on his time, as well as his poor mental and physical health, combined with his over-availability to others took their toll on Kohlberg and Lucille’s marriage (Kurtines & Gewirtz, 1991; Snarey, 2012; Springer, 2005; Zizek et al., 2015).

This reduced the effectiveness of the proximal processes, leading to a dysfunctional outcome in their marriage (Bronfenbrenner & Morris, 2006). In addition, Kohlberg’s parents’ divorce in his childhood and adolescence years, could have further influenced Kohlberg’s interactions and perceptions within his own marriage to Lucille (Fowler et al., 1988). Although there was limited literature on the impact of Kohlberg’s separation and divorce from Lucille, Helkama recalled that Kohlberg seemed disorientated due to their separation (Zizek et al., 2015).

The second outcomes area related to Kohlberg’s mental health within his success years was his **career**. Between the years of 1968–1970, his proximal processes in the academic sphere were functioning at an optimal level (Bronfenbrenner & Morris, 2006). However, due to Kohlberg’s chronic *giardiasis*, the efficiency of his proximal processes from 1971/1972 onwards was challenged. He often had to excuse himself from meetings and gradually developed the resource characteristics of being unreliable at work (DeVries, 1991; Higgins, 1991; Snarey, 2012; Springer, 2005).

Demonstrating the impact of Kohlberg’s poor health on his work, before his death, Kohlberg’s physical pain was so extreme, that he could no longer focus on his work and function effectively. These accounts pointed to the deterioration of Kohlberg’s once optimally functioning proximal processes. This led to a dysfunctional outcome in productivity levels within his career sphere, resulting in Kohlberg eventually handing over a lot of his own work to his colleagues and students (Bronfenbrenner & Morris, 2006; DeVries, 1991; Rest et al., 1988).

The third area considered in relation to Kohlberg’s mental health within the proximal processes during Kohlberg’s career success years stage was his **challenges versus protective factors**. While Kohlberg’s marriage to Lucille soured, he had the protective factors of his love for his work within his mesosystem and exosystem, as well as his friendships with his colleagues, students and friends within his microsystem. After Kohlberg’s marriage to Lucille ended, he also had the protective factor of his relationship with Ann Higgins within his microsystem (Higgins, 1991; Snarey, 2012; Springer, 2005; Walsh, 2000). These protective factors assisted Kohlberg to achieve a competence outcome with some of his interpersonal relationships (Bronfenbrenner & Morris, 2006).

One important area where protective factors failed to mitigate Kohlberg’s challenges was with his physical health. In the last few years of Kohlberg’s life, he tried to find a cure for his giardiasis, after modern medicine failed to help him. This was evident when Kohlberg travelled to Finland after reading that the Finnish doctors had come up with a new treatment for giardiasis. Another example was Kohlberg experimenting with different unconventional remedies (Springer, 2005; Zizek et al., 2015). In addition, despite Kohlberg’s ailing health, he continued working to a relentless work schedule (Fowler et al., 1988). Kohlberg found it difficult to take time off work to recover.

As a result, there was a complementary relationship between Kohlberg’s deteriorating health and his levels of productivity at work. While his proximal processes once functioned optimally within his vocation, his deteriorating health and mental health challenges eventually led to an outcome of dysfunction with Kohlberg’s productivity levels (Bronfenbrenner & Morris, 2006). Despite the deterioration in Kohlberg’s productivity levels, the historical context in the USA served as a protective factor for Kohlberg reputation. Rest et al. (1988) explained that the social, political and cultural atmosphere in the USA within his macrosystem increased Kohlberg’s popularity. These protective factors assisted in maintaining competence outcomes within Kohlberg’s career (Bronfenbrenner & Morris, 2006).

The final theme related to Kohlberg’s mental health was his **visionary qualities**. Kohlberg showed visionary qualities in his development of a seventh stage in his theory of moral development, referred to as the metaphysical stage (Higgins, 1991; Zizek et al., 2015). The seventh stage provided him with comfort as it answered questions such as ‘Why live?’ (Higgins, 1991; Zizek et al., 2015). These activities gave Kohlberg’s proximal processes a competence outcome within this area (Bronfenbrenner & Morris, 2006).

### Mental Health

Although the information regarding Kohlberg’s mental health was limited, this area is important, as his mental health probably played an important aspect in the tragic ending to his life. During Kohlberg’s childhood, there is no mention of his mental health. During Kohlberg’s time with the Haganah forces, the literature implied that he might had experienced mental health difficulties. This was apparent when Kohlberg’s friend Gutmann, who met Kohlberg when they served together on the Haganah boat for a year, revealed that Kohlberg had suffered from mood swings throughout the years (Langley, 1987).

Despite this revelation, the literature did not bring to light mental health difficulties during Kohlberg’s early career substage within his career establishment years. This could mean that during Kohlberg’s time with the US Marines and the Haganah forces, his possible mental health issues were situational, due to the inherent stress and anxiety from seeing the devastation from WWII and from breaking international law by smuggling refugees into Palestine (DeVries, 1991; Higgins, 1991; Snarey, 2012). However, the suggestion from Gutmann that Kohlberg suffered from mood swings throughout the years (Langley, 1987) could signify that his mental health challenges were present prior to him contracting chronic giardiasis.

However, Kohlberg’s mental health was not specifically discussed in the literature until he contracted *giardiasis* (DeVries, 1991; Higgins, 1991; Snarey, 2012). The deterioration of Kohlberg’s physical health, and his separation and subsequent divorce from Lucille worsened his mental health (Snarey, 2012; Springer, 2005). Perpetuating Kohlberg’s poor mental health was his disinclination to take a break from work or to ease his workload so that he could recover (Fowler et al., 1988; Zizek et al., 2015). Further exasperating Kohlberg’s mental health difficulties was that he endured physical and mental anguish in silence (Walsh, 2000).

The authors’ experience is that often society holds gifted or extraordinary individuals to unrelenting standards with their mental health. In Kohlberg’s case, society’s high expectations were perpetuated further as he was also working within the helping vocation. Society often perceives individuals in helping professions to be able to efficiently attain holistic well-being due to their specialised knowledge and training. Perhaps this was why Kohlberg continued with a demanding work schedule, thereby allowing him to maintain the image of functioning optimally despite his difficulties (Botes, 2022). This corresponds with Kohlberg’s friends and family being angry with him for committing suicide and feeling guilty for not being able to help him. An example of this was when his friends and family stated, “if only we would have been more sensitive to his state of mind, perhaps it would have never led to this” (Fowler et al., 1988, p. 219).

### Conclusion

With the increased rates of suicidal ideation and suicide due to the lasting impact of Covid-19 on society (high unemployment rates, new mental health challenges and social isolation) (Substance Abuse and Mental Health Services Administration, 2023), this article highlights the importance of suicide awareness through destigmatizing suicide and emphasising the importance of approaching mental health challenges through a holistic approach in overcoming crises.

The psychobiographical analysis of Kohlberg in the last stage of his life, using Bronfenbrenner’s BTHD Framework (Bronfenbrenner & Morris, 2006) illustrated the importance of exploring the complexities and multi-layered factors involved in understanding motivating factors which increase chances of suicide, as well as protective factors which could mitigate suicidal ideation and intent. Understanding the complexities involved in factors increasing the risk of suicide allows for challenges to be addressed and worked through more thoroughly to attain greater equilibrium in overall mental health well-being (Botes, 2022).

This study endeavoured to explore the utility in Bronfenbrenner’s BTHD in offering an intricate view of the nuances and complexities involved in understanding suicidality. By exploring the complex interactions between Kohlberg’s characteristics, environmental influences, family origins and historical context, this psychobiography illustrated how these interactions contributed to the functioning of his proximal processes and eventual suicide. Thus, this study demonstrated the helpfulness of this theoretical approach in offering a more interconnected understanding of factors associated with Kohlberg’s emotional deterioration and suicide. This psychobiography also contributes to research focused on Bioecological factors influencing the mental health and suicidality in high-performing individuals.

## Data Availability

Primary and secondary sources available in the public domain.
